# Sleep Duration Is Closely Associated with Suicidal Ideation and Suicide Attempt in Korean Adults: A Nationwide Cross-Sectional Study

**DOI:** 10.3390/ijerph18115594

**Published:** 2021-05-24

**Authors:** Yujin Ko, Jieun Moon, Sangsoo Han

**Affiliations:** 1 Department of Psychiatry, Soonchunhyang University Bucheon Hospital, 170 Jomaru-ro, Bucheon 14584, Korea; aprilujin@gmail.com; 2 Department of Biostatistics, Clinical Trial Center, Soonchunhyang University Bucheon Hospital, 170 Jomaru-ro, Bucheon 14584, Korea; moon6188@schmc.ac.kr; 3 Department of Emergency Medicine, Soonchunhyang University Bucheon Hospital, 170 Jomaru-ro, Bucheon 14584, Korea

**Keywords:** cross-sectional studies, sleep, suicide attempts, suicidal ideation

## Abstract

Introduction: Suicidal ideation and suicide attempts are major risk factors for suicidal death, and sleep problems are associated with an increased risk for mental disorders. We investigated the relationship between sleep duration and suicidal ideation and suicide attempts in a representative sample of the Korean general population from a nationwide survey. Methods: We analyzed data collected from the Korea National Health and Nutrition Examination Survey VI and VII (2013–2018). Suicidal ideation was identified via self-report, and we accessed suicide attempt history. Sleep duration was divided into three categories: short sleep duration (SSD) (≤5 h), normal sleep duration (NSD) (>5 and <9 h), and long sleep duration (LSD) (≥9 h). Sampling weights were applied to obtain estimates for the general Korean population. Results: Overall, 4015 (12.0%), 25,609 (76.5%), and 3857 (11.5%) participants were in the SSD, NSD, and LSD groups, respectively. Among these groups, 7.2%, 2.8%, and 3.3% reported suicidal ideation; while 1.2%, 0.4%, and 0.7% reported a history of suicide attempts. Multiple regression analyses revealed that SSD was significantly more strongly associated with suicidal ideation (adjusted odds ratio (AOR) 1.46, *p* < 0.001) and attempts (AOR 2.05, *p* = 0.003) than NSD. No association was found between LSD and suicidal ideation/attempts. Conclusion: Sleep duration is significantly associated with suicidal behavior, and SSD was particularly closely related with an increased risk for suicidal ideation and suicide attempt. Clinicians should carefully consider sleep duration in suicidal patients.

## 1. Introduction

Suicide is a major health problem worldwide, with nearly one million deaths to suicide per year [1]. In Korea, the suicide rate was 28.6 per 100,000 people in 2019, ranking first among developed countries, and it was the fourth cause of death after cancer, cardiovascular disease, and cerebrovascular disease [2]. Suicidal ideation and suicide attempts are strong factors for suicide deaths, and they can result in injuries and hospitalizations that represent a financial societal burden of billions of dollars [3]. Therefore, investigating the risk factors for suicidal ideation and attempts is considered an important element of formulating suicide prevention strategies.

Sociocultural, environmental, psychological, and biological factors contribute to suicidal ideation and suicide attempts [4,5,6]. Recently, sleep disorders such as insomnia and excessive sleep have been reported to be risk factors for suicidal ideation and suicide attempts [7]. Sleep is essential for health and well-being, including cognitive abilities, physiological processes, emotional regulation, physical development, and quality of life [8]. Sleep is also known to be closely related to depression and mental disorders such as bipolar disorder and anxiety disorder [9]. Previous studies have reported that a short sleep duration is associated with a heightened suicide risk [7,10,11]. However, whether sleep duration and suicidality exist independently of other mental disorders is unclear, and the direct mechanisms underlying a potential causal relationship between sleep time and suicide have not been identified [12].

Although multiple factors such as socioeconomic environment and mental health problems are associated with suicide, few studies have investigated the relationship between sleep duration and suicidal ideation after correcting for depression, health status, and sociodemographic factors in the general adult population in Asia [13,14]. One previous study that did correct for these factors found an association between sleep duration and suicidal ideation in Korean adults [11]. However, that study did not consider suicide attempts, which, along with suicidal ideation, are a major risk factor for suicide.

To address this, we conducted a cross-sectional study using a large-scale sample representing Korean adults. We corrected for potential confounders such as depressive mood, health status, and sociodemographic factors to comprehensively evaluate the association between sleep duration and suicidal ideation and suicide attempts.

## 2. Materials and Methods

### 2.1. Study Population and Sampling

We analyzed data collected between 2013 and 2018 as part of the Korea National Health and Nutrition Examination Survey (KNHANES) version VI (2013–2015) and VII (2016–2018). The Korea Centers for Disease Control and Prevention (KCDC) conducts annual surveys to evaluate the health and nutritional status of Korean families. This national, cluster, multi-level, stratified survey has a random sampling method, and it has been proportionally distributed by region, sex, and age in the Korean population since 1998. Survey participants vary from year to year and are not continuously monitored. There are three main components: a health interview survey, a health examination survey, and a nutrition survey. Participants under 19 years of age, and those who did not complete the sleep duration survey were excluded from the present study.

### 2.2. Definition of Sleep Duration, Suicidal Ideation, and Suicide Attempts

Sleep duration was identified using self-reported data, specifically, responses to the question “How many hours do you usually sleep a day?” The participants were categorized as having a short sleep duration (SSD) (≤5 h), normal sleep duration (NSD) (>5 and <9 h), or long sleep duration (LSD) (≥9 h) according to the guidelines of the National Sleep Foundation [8].

The individuals who answered “yes” to the question “Have you ever seriously considered suicide in the last year?” were defined as having suicidal ideation. Those who answered “yes” were asked if they had attempted suicide. Questions asking suicidal ideation and history of suicide attempts have been previously used in other surveys to detect suicide risk [15].

### 2.3. Description of Demographic Variables

The demographic characteristics, socioeconomic status, and personal history including the medical and lifestyle habits of the participants were examined via health examinations and interviews. Diagnoses of any major comorbidity such as high blood pressure, diabetes, dyslipidemia, stroke, ischemic heart disease (myocardial infarction, angina), asthma, and malignant tumor (lung, stomach, liver, colon, breast, uterine, or cervical) were assessed for all participants.

Body mass index (BMI) was determined for each participant as weight divided by height squared, and obesity was defined as BMI ≥25.0 kg/m^2^. Smoking status was classified as nonsmoker, ex-smoker, and current smoker. Alcohol habit was categorized based on the frequency of drinking alcohol: none, ≤1 drink/month, 2 drinks/month to 3 drinks/week, and ≥4 drinks/week. Educational level was categorized as ≤6 years (elementary school), 7 to 9 years (middle school), 10 to 12 years (high school), and ≥13 years (college or university). Household income level was divided into quartiles. Occupations were unemployed, simple labor (e.g., technicians and low-level laborers), agriculture, fishery, sales and services, and office workers. Physical activity was defined as follows: mid-intensity physical activity for at least 2 h 30 min per week, high-intensity physical activity for >1 h 15 min, or a combination of middle and high-intensity physical activity for a greater time period than stated above (1 min of high-intensity activity was defined as 2 min of mid-intensity activity) [16].

Marital status was single, married, separated, divorced, and separated by death. Perceived health status was examined via a question regarding how each individual felt about his or her health, i.e., “How do you perceive your own health?” The responses were categorized as follows: very good, good, average, bad, and very bad. Depressive mood was assessed via a “yes” or “no” answer to the question “In the past year, have you ever been depressed or desperate enough that it interfered with your daily life for more than 2 weeks?”

### 2.4. Statistical Analysis

We used IBM SPSS Statistics for Windows, version 26.0 (IBM Corp., Armonk, NY, USA) for statistical analyses. We used the analysis of variance to compare the continuous variables and the chi-square test for the categorical variables. Multiple logistic regression analyses were performed to confirm that the difference caused by the confounding variable was not due to sleep duration. To this end, we used the following three different models; model 1, unadjusted odds ratio; model 2, adjusted by age and sex; model 3, fully adjusted by age, sex, and other environmental factors such as smoking, alcohol consumption, educational level, household income, occupation, physical activity, sleep duration, marital status, perceived health status, depressive symptoms, and comorbidities. Sampling weights were applied in the analyses so that the estimates were representative of the Korean population. Statistical significance was defined as *p* < 0.05.

## 3. Results

**Figure 1:**
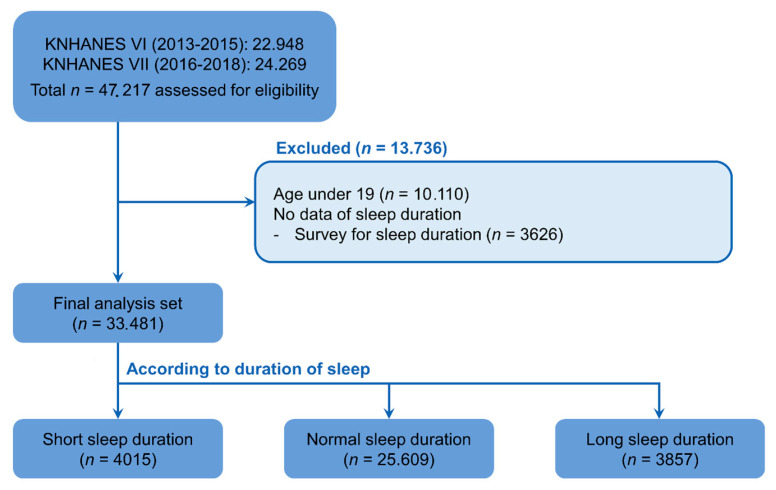
Flow chart of study subjects included in the 2013 to 2018 Korea National Health and Nutrition Examination Surveys (KNHANES VI and VII).

A total of 42,217 people participated in the KNHANES examination and health survey during the study period. Excluding those under the age of 19 (10,110 people) and those who did not complete the sleep duration survey (3626 people), data from 33,481 people were included in the analyses (Figure 1).

### 3.1. Demographics of Participants According to Sleep Duration

Of the 33,481 participants in the study, 4015 (12.0%) people had an SSD, 25,609 (76.5%) had an NSD, and 3857 (11.5%) had an LSD. The sleep duration in each group was 4.61, 7.03, and 9.41 h, respectively. Overall, 1208 (3.6%) people reported suicidal ideation. When classified by sleep status, 287 (7.2%) in the SSD, 704 (2.8%) in the NSD, and 127 (3.3%) participants in the LSD group reported suicidal ideation. In terms of suicide attempts, 187 (0.6%) people reported attempting suicide, of which 49 (1.2%) were from the SSD, 110 (0.4%) were from the NSD, and 28 (0.7%) were from the LSD group. Suicidal ideation and suicide attempts were the most common in the SSD group (*p* < 0.001). Age, sex, height, weight, alcohol consumption, educational level, occupation, household income, physical activity, marital status, perceived health status, depressive symptom, and comorbidities were also significantly different between the three groups (*p* < 0.001) (Table 1).

### 3.2. Association between Suicidal Ideation and Sleep Duration

**Figure 2:**
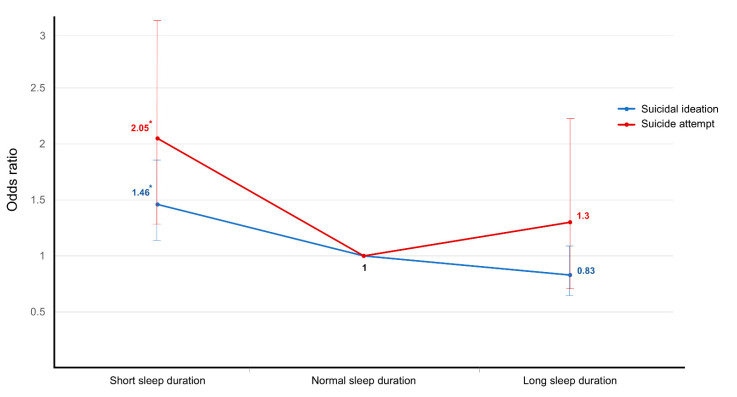
Association between sleep duration and suicidal ideation and suicide attempts. The logistic regression model was fully adjusted in terms of age, sex, and other environmental factors such as smoking, alcohol consumption, educational level, household income, occupation, physical activity, duration of sleep, marital status, perceived health status, depressive symptoms, and comorbidities. * *p* < 0.05.

The estimated odds ratios of suicidal ideation and sleep duration from the multiple logistic regression analyses are presented in Table 2. In model 1, which was unadjusted, the odds of suicidal ideation in participants in the SSD group were 2.67 (95% confidence interval (CI) 2.27–3.14, *p* < 0.001) times greater than that in the NSD group. In model 2, which was adjusted by age and sex, the odds of suicidal ideation in the SSD group were 2.46 times higher than that in the NSD group (95% CI 2.08–2.91, *p* < 0.001). In model 3, which was fully adjusted by age, sex, and other environmental factors such as smoking, alcohol consumption, educational level, household income, occupation, physical activity, duration of sleep, marital status, perceived health status, depressive symptoms, and comorbidities, the odds of suicidal ideation in the SSD group were significantly higher than those in the NSD group (OR 1.46, 95% CI 1.18–1.81, *p* < 0.001) (Figure 2). The LSD group did not show any significant differences among the data calculated using the three models for suicidal ideation.

### 3.3. Association between Suicide Attempts and Sleep Duration

The results of the multiple logistic regression analyses for suicide attempts and sleep duration are shown in Table 3. The SSD group showed a more strongly positive association with suicide attempts than the LSD group. In model 3, the rate of suicide attempts in the SSD group was 2.05 times higher than that in the NSD groups (95% CI 1.18–3.26, *p* = 0.003), and the odds ratio was significantly higher than that of suicidal ideation (20.5 vs. 1.46) (Figure 2). The LSD group was not statistically significantly related to suicide attempts compared to the NSD group.

## 4. Discussion

In this nationwide cross-sectional study based on a representative sample of the general population in Korea, we investigated the association between sleep duration and suicidal ideation and suicide attempts. We found that SSD (≤5 h) is an independent risk factor for suicidal ideation and suicide attempts.

When faced with suicidal patients, clinicians tend to focus on identifying events or stressors that may have triggered suicidal behaviors and treating any mood symptoms such as depression or anxiety. However, our data indicate that sleep duration, particularly SSD, is one of the strongest risk factors for suicide attempts, as well as suicidal ideation. This finding is consistent with previous studies, which have shown a U-shaped relationship between sleep duration and suicidal risk [10,11,17], where suicidal risk is higher in short sleepers. In addition, a recent study reported that LSD is related to suicidal ideation and that SSD is related to suicidal behaviors in general [18]. Therefore, clinicians should be aware that sleep duration is closely related with suicidality and thus carefully monitor sleep duration when treating suicidal patients.

Sleeping for a normal duration is necessary to regulate body functions and resources that are depleted during the day and plays an important role in the functional recovery of the central nervous system, which is essential for maintaining overall health [8]. However, insufficient sleep can lead to various psychological and physiological disorders, including impaired judgment, decreased concentration, and poor impulse control, along with endocrine and immunological changes [19,20]. Although the exact mechanism of the association between sleep disturbance and suicidal tendencies has not been clearly elucidated, it may be related to the inhibition of 5-hydroxytriptamine [21,22]. In addition, inflammation markers, particularly interleukin-6, may be associated with suicidal risk in patients with sleep disorders [17]. As we did not explore the mechanisms linking sleep and suicide risk in the present study, additional clinical trials are needed.

Treatment for sleep disturbance can be crucial for reducing the risk of suicide. Previous studies have also investigated treatment effects of insomnia on reducing suicide risk and mentioned the importance of sleep management [23,24]. The causes for sleep disturbance are heterogenous for each individual, and the treatment also varies depending on the cause. The importance of cognitive–behavioral therapy for insomnia (CBT-I) and treatment for the comorbid disease is emphasized in many studies, and medications are also recommended if necessary [25]. CBT-I has been proven to mitigate suicidal risk [26]. Sleep medicines have shown mixed results, but growing evidence suggests its efficacy for sleep disturbance in people who are depressed or have nightmare problems [24]. As the American Academy of Sleep Medicine suggests, the decisions should be made based on clinical experience, past drug reactions, personal preferences, and side effects all together [27].

Low socioeconomic status, vulnerable health status, disability, and depression are well-known risk factors for suicide, and other factors such as marital status and family history have also been reported [28,29]. An epidemiological survey of mental health conditions which was conducted nationwide in Korea indicated that mental disorders, alcohol consumption, and smoking were related to suicidal behaviors [30]. In this study, we adjusted not only for socioeconomic factors, marital status, health behavior, and health status but also for depressive symptoms. We found that the odds ratio of suicidal ideation and suicide attempts in the SSD group gradually decreased as we increased the number of adjustments. This suggests that suicidal risk can be mediated by these factors.

Previous studies have reported that there are factors distinguishing those with suicide attempts from those who have experienced suicidal ideation but never attempted suicide. A markedly high presence of depressive disorder or hopelessness was seen in suicide ideators, and suicide attempters could be distinguished by a higher prevalence of comorbid substance use disorder, a younger age of onset, and a lack of social support [31,32]. The capability to attempt suicide caused progression from ideation to attempts [3]. In this study, SSD was found to be a common factor for both suicidal ideation and attempts, and we did not distinguish these two.

The major strength of this study is the large, population-based dataset, which enabled us to evaluate the relationship between sleep duration and suicidal ideation and suicide attempts in the general population. Our results are consistent with those of previous studies on the relationship between sleep duration and suicidality conducted in Korea and China [10,11]. Therefore, our findings can potentially be generalized not only to the Korean population but also to general Asian populations. To the best of our knowledge, this is the largest study to examine the association between sleep duration and suicidal ideation and suicide attempts in an Asian population.

However, some limitations must be considered. First, this study had a cross-sectional design and used data from a national health survey. Therefore, we were only able to evaluate simple correlations and thus did not examine the causal relationship between sleep quantity and suicidal ideation and suicide attempts. Second, the relationship between sleep duration and suicide may vary depending on ethnicity and race. Since the KNHANES survey is based on the general population of Korea, care should be taken when applying findings from this dataset to other population groups. Third, we could not analyze factors that were not included in the KNHANES survey. Additional information on insomnia and other comorbid diseases such as sleep apnea were not included in the survey so the effects of these factors could not be considered. Finally, we were unable to evaluate the mechanisms underlying the relationship between sleep and suicide risk. Therefore, a future well-designed large-scale study is needed to overcome these limitations.

## 5. Conclusions

Sleep duration was strongly associated with suicidal ideation and suicide attempts. Specifically, suicidal ideation and suicide attempts were significantly more frequent in people who had fewer hours of sleep, and short sleep duration was particularly strongly associated with suicide attempts. As such, given that sleep duration can be considered an independent and strong risk factor for suicidality, treating sleep disturbances can be considered as a means of reducing suicidal behavior risk.

## Figures and Tables

**Table 1:** Baseline characteristics according to sleep duration for the study population.

Variables	SSD	NSD	LSD	*p*-Value
	(*n* = 4015)	(*n* = 25,609)	(*n* = 3857)	
Age, yrs	56.8 ± 16.27	49.97 ± 16.19	52.18 ± 19.52	<0.001
Sex, *n* (%)				<0.001
Male	1478 (36.8)	11,396 (44.5)	1527 (39.6)	
Female	2537 (63.2)	14,213 (55.5)	2330 (60.4)	
Height, cm	160.15 ± 9.75	163.25 ± 9.22	161.41 ± 9.27	<0.001
Weight, kg	62.6 ± 12.48	63.78 ± 12.2	61.79 ± 11.93	<0.001
BMI, kg/m^2^	24.3 ± 3.63	23.84 ± 3.47	23.66 ± 3.68	<0.001
Obesity, *n* (%)	1534 (38.2)	8575 (33.5)	1221 (31.7)	<0.001
Smoking status, *n* (%)				0.186
Non-/Ex-smoker	3278 (81.6)	20,828 (81.3)	3184 (82.6)	
Current smoker	737 (18.4)	4781 (18.7)	673 (17.4)	
Alcohol consumption, *n* (%)				<0.001
None	1484 (37.0)	6704 (26.2)	1318 (34.2)	
≤1 drink/mo	981 (24.4)	7333 (28.6)	1045 (27.0)	
2 drinks/mo to 3 drinks/wk	1204 (30.0)	9947 (38.8)	1195 (31.0)	
≥4 drinks/wk	346 (8.6)	1625 (6.4)	299 (7.8)	
Education level, *n* (%) ^†^				<0.001
≤6 y	1465 (36.8)	4747 (18.4)	1161 (30.2)	
7–9 y	498 (12.1)	2617 (9.9)	458 (11.7)	
10–12 y	1147 (28.6)	8677 (34.1)	1156 (30.0)	
≥13 y	905 (22.5)	9568 (37.6)	1082 (28.1)	
Occupation, *n* (%)				<0.001
Unemployed (student, housewife, etc.)	1905 (48.1)	9534 (37.6)	1940 (50.8)	
Office work	640 (15.8)	6650 (26.1)	601 (15.5)	
Sales and services	472 (11.6)	3416 (13.2)	445 (11.4)	
Agriculture, forestry, and fishery	529 (13.0)	3798 (14.7)	533 (13.7)	
Machine fitting and simple labor	469 (11.5)	2211 (8.4)	338 (8.6)	
Household income, *n* (%) ^‡^				<0.001
Low	1190 (29.7)	4178 (16.3)	1084 (28.1)	
Low-moderate	1052 (26.2)	6210 (24.3)	1016 (26.4)	
Moderate-high	915 (22.8)	7258 (28.4)	987 (25.6)	
High	858 (21.4)	7963 (31.1)	770 (20.0)	
Physical activity, *n* (%)	1024 (26.2)	8248 (32.9)	793 (20.9)	<0.001
Duration of sleep, h	4.61 ± 0.7	7.03 ± 0.78	9.41 ± 0.72	<0.001
Marital status, *n* (%)				<0.001
Single	461 (11.5)	4274 (16.7)	735 (19.1)	
Married	2551 (63.5)	18,256 (71.3)	2514 (65.2)	
Separated	37 (0.9)	153 (0.6)	20 (0.5)	
Separated by death	709 (17.7)	1917 (7.5)	453 (11.7)	
Divorced	257 (6.4)	1009 (3.9)	135 (3.5)	
Perceived health status, *n* (%)				<0.001
Very good	200 (4.6)	1263 (4.7)	179 (4.5)	
Good	719 (17.9)	6613 (25.9)	815 (21.1)	
Average	1916 (47.9)	13,224 (52.2)	1909 (49.8)	
Bad	855 (21.3)	3569 (13.8)	713 (18.5)	
Very bad	325 (8.3)	940 (3.4)	241 (6.1)	
Depressive symptom, *n* (%)	443 (11.0)	1381 (5.4)	272 (7.1)	<0.001
Suicidal ideation, *n* (%)	287 (7.2)	704 (2.8)	127 (3.3)	<0.001
Suicide attempt, *n* (%)	49 (1.2)	110 (0.4)	28 (0.7)	<0.001
Comorbidities, *n* (%)				
Hypertension	1268 (31.6)	5508 (21.5)	1076 (27.9)	<0.001
Diabetes	505 (12.6)	2127 (8.3)	444 (11.5)	<0.001
Dyslipidemia	871 (21.7)	3977 (15.5)	642 (16.7)	<0.001
Stroke	131 (3.3)	529 (2.1)	148 (3.8)	<0.001
Myocardial infarction	51 (1.3)	242 (0.9)	50 (1.3)	0.033
Angina	122 (3.0)	445 (1.7)	93 (2.4)	<0.001
Asthma	166 (4.1)	707 (2.8)	143 (3.7)	<0.001
Malignancy	88 (2.2)	427 (1.7)	76 (2.0)	0.038

Numeric parameters are expressed as mean ± standard deviation and categorical parameters are expressed as counts and percentages in parentheses. SSD, short sleep duration; NSD, normal sleep duration; LSD, long sleep duration; BMI, body mass index. ^†^ Educational level was categorized into the following four groups: ≤6 years (elementary school), 7–9 years (middle school), 10–12 years (high school), and ≥13 years (college or university). ^‡^ Household income level was measured at the level when compared with the standard amount for each age, and then it was was grouped into quartiles.

**Table 2:** Association between sleep duration and suicidal ideation.

	Model 1			Model 2			Model 3		
	OR	95% CI	*p*-Value	OR	95% CI	*p*-Value	OR	95% CI	*p*-Value
SSD	2.67	2.27–3.14	<0.001	2.46	2.08–2.91	<0.001	1.46	1.18–1.81	<0.001
NSD	1			1			1		
LSD	1.12	0.90–1.40	0.301	1.10	0.88–1.37	0.398	0.83	0.64–1.08	0.166

OR, odds ratio; CI, confidence interval; SSD, short sleep duration; NSD, normal sleep duration; LSD, long sleep duration. Model 1 was the unadjusted odds ratio. Model 2 was adjusted by age and sex. Model 3 was fully adjusted by age, sex, and other environmental factors such as smoking, alcohol consumption, educational level, household income, occupation, physical activity, duration of sleep, marital status, perceived health status, depressive symptoms, and comorbidities.

**Table 3:** Association between sleep duration and suicide attempts.

	Model 1			Model 2			Model 3		
	OR	95% CI	*p*-Value	OR	95% CI	*p*-Value	OR	95% CI	*p*-Value
SSD	3.65	2.56–5.20	<0.001	3.74	2.59–5.39	<0.001	2.05	1.28–3.26	0.003
NSD	1			1			1		
LSD	1.56	0.98–2.48	0.062	1.48	0.92–2.37	0.105	1.30	0.75–2.28	0.349

OR, odds ratio; CI, confidence interval; SSD, short sleep duration; NSD, normal sleep duration; LSD, long sleep duration. Model 1 was the unadjusted odds ratio. Model 2 was adjusted by age and sex. Model 3 was fully adjusted by age, sex, and other environmental factors such as smoking, alcohol consumption, educational level, household income, occupation, physical activity, duration of sleep, marital status, perceived health status, depressive symptoms, and comorbidities.

## Data Availability

The data files are available from the KCDC and Prevention database on the following webpage: https://knhanes.cdc.go.kr/knhanes/sub03/sub03_02_05.do (accessed on 12 April 2021). Anyone who wishes to check the data can access the web page and receive the raw data after meeting appropriate qualifications.
